# New drug regulations in France: what are the impacts on market access? Part 2 – impacts on market access and impacts for the pharmaceutical industry

**DOI:** 10.3402/jmahp.v1i0.20892

**Published:** 2013-08-06

**Authors:** Cécile Rémuzat, Mondher Toumi, Bruno Falissard

**Affiliations:** 1 Creativ-Ceutical, Paris, France; 2 Department of Decision Sciences and Health Policies, University Claude Bernard of Lyon 1, UFR d'Odontologie, Lyon, France; 3 INSERM Unit U669 (Public Health and Mental Health), Maison de Solenn, University Paris-Sud, Paris, France

**Keywords:** France, market access, drugs, law, pricing, reimbursement, comparative evidence, medico-economic assessment

## Abstract

Access to the French drug market is being impacted by an ongoing dramatic shift in practice as well as by two laws that came into force in December 2011. This new environment has been described and analyzed in two separate articles. This second article analyzes how this new environment will actually impact the access to French drug market. French drug market access will be increasingly driven by comparative-effectiveness and cost-effectiveness data, and an increased role of postmarketing studies in the years to come. This access is evolving in a more complex environment for stakeholders due to the uncertainties surrounding these changes and it will be more complex and difficult for the pharmaceutical industry to address. The main issue faced by the pharmaceutical companies will be to minimize uncertainty at the time of a drug's launch to narrow the decision window. This is a major change of paradigm for the pharmaceutical business, in which pre- and postlaunch risks are directed toward the pharmaceutical industry.

The current process of access to the French drug market is experiencing a shift in its health technology assessment (HTA) practice, although this has never been officially reported; and the process will be impacted by further changes in the years to come, following two bills that were passed in 2011: the law for the reinforcement of the health safety of drug and health products (1), and the Social Security Funding Law for 2012 (2). The objective of this article is to analyze these recent changes in access to French drug market. The description of these changes is presented in a separate article, ‘New Drug Regulations in France: What Are the Impacts on Market Access? Part 1 – Overview of New Drug Regulations in France’.

## Impact of the new French drug regulations on market access

In light of the recent changes in drug regulations, it is evident that access to French drug market will be increasingly driven by comparative-effectiveness and cost-effectiveness data, and an increased role of postmarketing studies in the years to come.

### Comparative evidence and real-world data

Comparative evidence and real-world data are pointed out as critical and as potentially conditioning the marketing authorization, and they will be increasingly requested.

Indeed, the National Agency for the Safety of Medicine and Health Products (Agence Nationale de Sécurité du Médicament et des Produits de Santé, or ANSM) has the option to require safety and efficacy postauthorization studies (in comparison with available reference treatments, when they exist). If the drug manufacturer or operator refuses to comply with the requirements of performing clinical trials versus active comparators, it has to justify it. Until now, in the context of European marketing authorization, comparative trials were requested by a member state in only a few cases and in the framework of the risk management plan. Such studies are also regularly requested by the Transparency Committee (Commission de la Transparence, or CT) and the Economic Committee on Healthcare Products (Comité Economique des Produits de Santé, or CEPS) for new drugs that enter into the market. The French National Authority for Health (Haute Autorité de Santé, or HAS) anticipates an increase in the requirement of such studies in relation to its new responsibility to follow up on appropriate drug usage. Studies to identify the actual target population, as well as effectiveness and potential misuse, are considered to be more consistently and systematically requested (Communication from the meeting HAS-AMIPS: The HAS in the new health environment in France and Europe, February 7, 2012). In such cases, the registration of a medicine on the list of reimbursable drugs will depend on the setup of clinical trials versus reference therapeutic strategies (when they exist). Moreover, the ANSM will reassess the benefit–risk ratio of already marketed products that could impact their marketing authorization and reimbursement level. Thus, it is anticipated that a product could be considered for reimbursement only if it produces head-to-head evidence, and these issues are very much overlapping between the HAS's and ANSM's field of responsibility. The parliamentary discussion on this topic was controversial as it confused a safety bill with reimbursement arguments, but the bill was finally adopted despite the risk of confusion and the controversy it has induced. During the discussion, article 14 had led to a debate on the bill regarding the setup of clinical trials versus ‘active comparators with the best Medical Benefit (Service Médical Rendu, or SMR)’, but this point had not been finally included in the law (3). This highlights the importance of comparative evidence, even though the application decree related to article 14 is still pending. It will be interesting to follow the application decree content and its implementation as many of today's most important innovations still reach the market without any active reference comparative trials. It is unlikely within the current French philosophy that such products will get halted; coverage with evidence development (CED) associated with an escrow agreement is likely to be the solution (4). It is interesting to note that the new contractual framework between the CEPS and the pharmaceutical companies (5) states that the CEPS can suggest a price conditional to the results of additional studies (mainly real-world studies) in case the improvement of medical benefit in terms of public health cannot be fully demonstrated by prelaunch clinical studies. Article 14 set up the legal grounds for CED. At the same time, a joint committee created by the HAS and CEPS for following up on real-world studies has been established.

### Medico-economic assessment

Cost-effectiveness studies will now be part of market access requirements for all drugs satisfying the selection criteria for medico-economic assessment. At the time of drug approval, the scope of assessment will be relatively narrow, and outstanding questions will be addressed during reassessments 5 years later or possibly earlier if warranted. Both HAS committees, the CT and the Economic and Public Health Assessment Committee (Commission Evaluation Economique et de Santé Publique, or CEESP), will continue to work independently. Health economics data will be taken into account by the CEPS, and the price setting will take into account the results of the medico-economic assessment performed by the CEESP. In October 2011, the HAS published a guideline related to methodological choices for economic assessments. This guideline remains very flexible even if some specific recommendations were made (e.g., no cost–benefit analysis, loss-of-productivity data are not included in reference cases, and deterministic and probabilistic sensitivity analysis is required). However, all options are possible as far as they are well argued and scientifically sound (6). The application decree related to the medico-economic missions of the HAS targets the budget impact, as well as the incremental benefit, as being the drivers for eligibility for medico-economic assessments of drugs. It has become obvious that the question of legislators was ‘How much to pay for a given added benefit?’. It is expected that health economics will help address that question, but the lack of clear reference cases will make it difficult to provide an answer. Although utility was clearly considered by the French Parliament (7), some resistance still remains from the CEESP to adopt it as a key reference measure of the benefit. CEESP will provide a ‘flash opinion’ that is expected not to impact decision timelines. Real-life health economic studies could be requested in the framework of the renewal of the inclusion of a drug in the formulary after its assessment, but observational economics studies might make it very difficult to generate the evidence due to multiple confounding factors and the sample size requested to provide evidence of a statistically significant difference.

This decree creates confusion and conflicting information with respect to current regulation and practice:

1. It can be seen that some of the HAS recommendations on the eligibility criteria for the medico-economic assessment of drugs were not implemented in the decree, with remaining areas of uncertainties such as the medico-economic assessment of orphan drugs or of drugs with minor ‘Improvement of Medical Benefit’ (Amélioration du Service Médical Rendu, or ASMR) but with substantial budget impact (Communication from the meeting HAS-AMIPS. The HAS in the new health environment in France and Europe. Feb 7, 2012) (Table 1) (8).
2. Not taken into consideration in the drugs’ selection criteria for medico-economic assessment were the frequent cases for which a medico-economic assessment would be performed but not used for decision making. Indeed, companies claim ASMR level I, II, or III in a large proportion of cases, while only a few get such an outcome (e.g., in 2011 for a first drug assessment, 2 cases of ASMR I, II, or III were obtained, accounting for less than 1% of all ASMR) (8). Products that get ASMR level IV or V are not expected to have an impact on the pharmaceuticals budget. As such, if a product is claimed to be an ASMR I, II, or III but is finally considered an ASMR IV or V by the CT, the medico-economic assessment already done will not be used for the decision making because this would be a waste of resources.
3. This new situation appears to conflict with the current regulation that is in force regarding the international reference pricing (versus the United Kingdom, Germany, Spain, and Italy) for products with ASMR I, II, or III. Although criticized, the reference pricing is still in force. The new contractual framework between the CEPS and the pharmaceutical companies (5) considers that the medico-economic assessment will modulate application of the international reference pricing with no specifications of how this will be implemented.
4. As both committees (CT and CEESP) operate independently, it is urgent that a clear process needs to be established to clarify a resolution between divergent opinions. While the CT focuses only on evidence-based clinical trials and officially on effectiveness, the CEESP is expected to have a broader perspective.
5. Finally, no information is available to specify how CEESP opinions will impact pricing negotiation.

Although it is not stated in any document, it would not be surprising if the CEPS will be tempted to use the recommended studies that are to be provided at the time of reassessment by CEESP as conditional studies for price setting (CED with or without an escrow agreement). Pharmaceutical companies can expect to have more difficulties going forward in obtaining the prices that they expected for their products, although for truly innovative products, this should still be possible. The complexity and hurdles to achieve premium pricing are expected to critically increase in regard to this concept.

**Table 1:** Suggestion of the HAS for the selection of drugs for medico-economic assessment and implementation status

Suggestions of selection criteria for medico-economic assessment of drugs	Implementation status
Claimed ASMR I, II, or III Except orphan drug status Except targeted population of patient < 1,000 persons	Only claimed ASMR I, II, or III implemented in decree no. 2012–1116
Claimed ASMR IV with budget impact estimated > €10 million	Not implemented in decree no. 2012–1116
Potential for expansion of the target population	Not implemented in decree no. 2012–1116
New mechanism of action	Not implemented in decree no. 2012–1116

## Other special considerations in terms of market access

### Difficulties in obtaining some experts’ advice

The new disposition regarding conflicts of interests could lead to difficulties in obtaining the advice of some experts. In some specialties, such as ophthalmology or rare diseases, the number of experts in France can be relatively limited (9). Moreover, there is a risk of delaying decisions. As an example, in the United States, the setting up of advisory committees of the Food and Drug Administration (FDA) to obtain independent expert advice on scientific, technical, and policy matters is delayed and incomplete in most cases, and this issue is ongoing in France; no later than December 2011, Director Dominique Maraninchi of the French Agency for the Medical Safety of Health Products (Agence Française de Sécurité Sanitaire des Produits de Santé, or AFSSAPS; currently known as ANSM) refused to publish, on behalf of AFSSAPS, the recommendations of the working group on anti-infectious treatments for upper respiratory tract infections due to several experts’ conflicts of interest with pharmaceutical companies. As a consequence, almost 40 experts resigned from this working group. Their work aimed to update the 2005 recommendations on systematic antibiotic treatment for upper respiratory tract infections in adults and children, and it has been consequently delayed (10).

### Limited access to off-label drugs

From now on, the off-label prescription rules will be binding for physicians and could restrict access to off-label drugs by patients as their reimbursement will be restricted. However, this will allow a better and more relevant control of off-label use. Agreements between the CEPS and the pharmaceutical companies can include some objectives to reduce off-label prescriptions, especially for public health reasons, with financial penalties in case of failure to meet these objectives (5).

### Increased uptake of generic drugs

Physicians have to prescribe drugs by their International Nonproprietary Name (INN) whenever possible, which might reinforce the prescription and dispensation of generic drugs.

### Decreased named-patient authorization for temporary use (autorisation temporaire d'utilisation, or ATU)

There has been a clear strengthening of access for named-patient ATU that should be an exception, and this was in line with the practice of AFSSAPS. The Parliament noticed that such drugs were priced freely and expressed concerns about their budget impact. Thus, this strategic approach will be limited from now on. The CEPS can require a reduction in the duration of the price guarantee, taking into account the ATU duration and the number of patients under ATU, or the reimbursement of all or a part of the expenses related to ATU (5). Although no condition is specified for such a reimbursement requirement, one could easily anticipate two conditions for reimbursement: the drug does not get an approval, or the price achieved after marketing authorization is far below the ATU price.

### Counteract monopolistic drug positioning

The extension of ‘Temporary Recommendation for Use’ (Recommandation temporaire d'utilisation, or RTU) to drugs having a therapeutic alternative will allow the use of medicines in some indications for which the pharmaceutical company refuses to request a marketing authorization.

### Other measures

Some other measures that appear new and important on the surface do not actually represent any true changes.

First, the new therapeutic index suggested by the HAS, which has not yet been implemented, is not expected to really impact the decisions of the CT as the decision criteria will remain quite the same, even if a new index could lead to a period of uncertainty regarding the reimbursement and pricing assessment rules. It should be noted that, according to the HAS chairman, the new mission of the French drug agency (as discussed in this article) led to the disappearance of the SMR notion, as a medicine with no medical benefit could not obtain a marketing authorization. This is quite inconsistent with the currently high level of approved products that still get scored with an insufficient SMR by the CT. It is unclear if all products approved for marketing will be recommended for reimbursement. It should be noted that the current methodology of the CT using the SMR–ASMR dichotomy is supported by the General Inspection of Social Affairs (Inspection Générale des Affaires Sociales, or IGAS), which recommends only some adjustments (11).

Then, from a safety perspective, all proposed measures of the new drug safety law were already part of the armament of AFSSAPS. What actually changed is that these measures are now explicit, and a clear emphasis on that specific role has been made by Parliament. So the safety measures are unlikely to dramatically change the practice.

## Impact for pharmaceutical industry

In light of these changes, it is apparent that access to French drug market will be increasingly challenging for the pharmaceutical industry. The main issue faced by the pharmaceutical companies will be to minimize the uncertainty at the time of a drug's launch to narrow its decision window (12). The main challenge will be to provide relative efficacy at the time of the launch and postmarketing comparative effectiveness, efficiency, and budgetary impact from field studies.

It is likely that CED will become the new facet of the French market access process for new innovative drugs, although this was historically dominated by price–volume agreements. Price–volume agreements will remain the cornerstone of the French system but will be complemented by CED.

These requirements will likely impose added burdens on companies in terms of cost and time. This can be a major hurdle: if the study design has to be endorsed ex ante, this will potentially impose complex negotiation; if discussed ex post, this might lead to non-acceptance of the study design. The level of hurdle imposed on a company to show evidence of medico-economic benefits in field research could easily become burdensome. Such studies might be unfeasible in many cases due to the very high sample size required. Indeed, due to multiple confounding factors, field data might never be able to show evidence of actual savings or resource utilization changes if studies are purely observational. However, control trials might be perceived as not fully representative of real life.

Modeling appears to be accepted as a first-line assessment at the time of launch to show the evidence of efficiency, incremental cost-effectiveness ratio (ICER), and budgetary impact. However, for postlaunch reassessment, authorities would like to see field studies confirming the model's provisions in term of effectiveness, ICER, and budgetary impact.

The lack of clear guidance on what could be requested and how it will be judged will add to the uncertainty of the French pharmaceutical market. Unlike the German market, the French reform does not provide, at that stage, a clear-cut, transparent, and reproducible methodology position paper for assessment of additional benefit or efficiency. In Germany, one could agree or disagree with the Institute for Quality and Efficiency in Health Care's (IQWiG) methodology, but it is very highly predictable for a given product.

A lot of uncertainty is inherent in the French culture, and exceptions are still the rule. For example, an ASMR V can still lead to a price premium (e.g., duloxetine, brand name Cymbalta^®^: ASMR V (March 14, 2007) (13), ex-factory price (28 tablets of 30 mg each): €16.52 (December 12, 2007) (14), and an insufficient SMR can still lead to 100% reimbursement [e.g., one drug in lung cancer (15)]. This shift in decision process without clear set rules for assessing the SMR and the ASMR shows that the decision analysis process is flexible and could change value judgment without new rules being established or communicated. Although the shift could be considered a reasonable decision, the issue is, as part of a process where public decision-making rules are changed with no communication and no stakeholder consultation, leading to uncertainty.

The application decrees of these new laws were expected to be issued before the presidential election (May 6, 2012), but this was not the case. This again increases uncertainty, as the new government has not yet issued all application decrees and might have different views on the market access policy for pharmaceuticals. Furthermore, the current majority belongs to the opposing party of the previous political majority and has expressed, through reports from the presidential office's advisor, serious reservations about current pricing and reimbursement policies (16, 17).

## Conclusion

French market access for pharmaceuticals will be more complex and more challenging to address for the pharmaceutical industry. It is evolving in a more complex environment for stakeholders due to the uncertainties surrounding these changes; even when the application decrees are issued, substantial uncertainty remains, as for example regarding medico-economic assessments. Areas of uncertainties need to be addressed in terms of postlaunch commitment requested by health authorities, therapeutic index implementation, and health economic assessment. In France, experience will set the new practice and rules ex post instead of defining ex ante the decision analysis framework as in England, Scotland, Sweden, Germany, and so on.

On top of price–volume agreements, CED will become more and more common as it is perceived by the committee as a good way to shift postlaunch uncertainty from the third-party payer to the pharmaceutical industry. It used to be the industry that carried prelaunch risk while the third-party payer carried the postlaunch risk. We are moving toward a system where the pre- and postlaunch risks are directed toward the same player: the pharmaceutical industry. This is a major paradigm shift for the pharmaceutical business.
